# Editorial: Mechanisms and Pathways Contributing to the Diversity of Aging Across the Tree of Life

**DOI:** 10.3389/fcell.2022.854700

**Published:** 2022-02-16

**Authors:** Alan A. Cohen, Joris Deelen, Owen R. Jones

**Affiliations:** ^1^ Department of Family Medicine, Research Centre on Aging, CHUS Research Centre, University of Sherbrooke, Sherbrooke, QC, Canada; ^2^ Max Planck Institute for Biology of Ageing, Cologne, Germany; ^3^ Cologne Excellence Cluster on Cellular Stress Responses in Ageing-Associated Diseases (CECAD), University of Cologne, Cologne, Germany; ^4^ The Interdisciplinary Centre on Population Dynamics (CPOP) and Department of Biology, University of Southern Denmark, Odense, Denmark

**Keywords:** aging, mechanisms, pathways, model organisms, evolution

## Introduction

The steadily increasing number of elderly individuals in our societies imposes an increased burden on our healthcare system due to an accompanying rise in chronic age-related diseases, including cardiovascular and neurodegenerative diseases (Niccoli and Partridge 2012). It is therefore of utmost importance to identify and understand mechanisms of aging across the tree of life and translate these discoveries into health-promoting interventions in humans (Partridge et al., 2018). Roughly 30 years ago, two important publications shaped our thinking on the evolution of aging and lifespan. Caleb Finch’s “Longevity, Senescence, and the Genome” (Finch 1990) provided an authoritative overview of what was known about the diversity of aging patterns across the tree of life, showing that some species appeared not to age. Kenyon and others (Kenyon et al., 1993) showed that simple genetic tweaks could dramatically extend lifespan in *Caenorhabditis elegans*, paving the way for an industry of research into conserved genetic pathways, driven by the idea of universal aging processes. Since then, the complexity and diversity of demographic aging patterns has been fleshed out (Jones et al., 2014; Jones and Vaupel 2017), while at the same time we are increasingly realizing that the mechanisms are also diverse (Cohen 2018). We are starting to move from a model of studying a few canonical model organisms (e.g. fruit flies and nematodes) complemented by a few exceptionally long-lived model organisms (e.g. naked mole rats, ocean quahogs, and hydra) to a need to characterize more systematically how mechanisms vary across species (Austad 2009). Beyond the basic science interest, this is essential from a translational perspective: a failure to understand the diversity of aging mechanisms among and within species, and how they interact, could be dangerous for proposed geroscience interventions. This Research Topic thus assembles cutting-edge work that sheds light on the mechanistic evolution of aging, from surveys of how single mechanisms vary across species to portraits of understudied species to eco-evolutionary perspectives and even novel theories of aging. Our hope is that these studies, presented together, will stimulate a new wave of thinking on how variation in mechanisms across species drives variation in aging, and on the underlying ecological, physiological, and evolutionary forces that may drive the mechanisms.

## Research Comparing Mechanisms Across Species

Our Research Topic contains several papers focusing on the conservation of aging mechanisms across species. These contain well-known mechanisms, such as mitochondrial dysfunction, protein toxicity and reactive oxygen species (ROS), but also understudied phenomena, such as reproductive suicide and eusociality. The review by van der Rijt and others (van der Rijt et al., 2020) focusses on mitochondrial dysfunction, one of the nine hallmarks of aging. The authors show that this hallmark is connected to many of the other hallmarks across the tree of life and argue that future studies on aging should thus take these interactions into account. Pras and Nollen (Pras and Nollen 2021) focused on another hallmark of aging, namely loss of proteostasis, and describe the different mechanisms responsible for maintaining a healthy proteome. They also provide examples of long-lived species that seem to have optimized protein homeostasis, showing that this hallmark is conserved across the tree of life. Oxidative damage, caused by ROS, is another mechanism often implicated in aging. The review by Shields and others (Shields et al., 2021) provides an overview of studies linking ROS and lifespan, showing the relationship between the two is complex, with ROS showing beneficial or detrimental effects depending on the species under study. Huang and others (Huang et al., 2021) focused their work on an understudied group of proteins in aging; flavin-containing monooxygenases (FMOs). They previously identified FMOs to be involved in lifespan regulation in *C. elegans* and now show that mammalian homologs affect stress resistance and metabolism in cellular models, which makes them an interesting target for further studies in model organisms and humans. Another understudied factor in aging is sociality and accompanying kin selection, which can modify selection pressures and the magnitude and direction of trade-offs (Bourke 2007). Increased sociality is associated with a longer lifespan (*see* (Arnold and Owens 1998; Thorley 2020) for examples) and eusociality, in particular, presents a fascinating window into the evolution of aging with greatly extended lifespans compared to non-social relatives, but also diversity in aging among castes (Keller and Jemielity 2006; Kramer et al., 2016). Giraldo and others (Giraldo et al., 2021) provide a mini-review examining the relationship between eusociality and brain senescence in eusocial species. They conclude that, though different taxa may show similarly extended lifespans and similar senescence phenotypes, the mechanisms involved are different.

## Novel Approaches to Study Aging Mechanisms

In addition, several papers in our Research Topic used novel approaches to study interactions between aging pathways and mechanisms. Simons and others (Simons et al., 2021), for example, showed that androgens do not mediate a simple trade-off between survival/maintenance and reproduction: relationships are often quadratic. Ukraintseva and others (Ukraintseva et al., 2021), on the other hand, looked at the interaction between genetic variants in genes involved in different aging-related pathways. They identified several combinations of genetic variants in genes implicated in different pathways that show an epistatic effect on survival to 85 years of age in two independent human studies.

## General Insights From Individual Species

Most articles in our Research Topic set their work in a comparative (cross-species) context, and some also highlight the value of undertaking detailed studies using non-canonical model organisms. For example, Yun (2021) provides a fascinating perspective on the regenerative abilities of salamanders. These exceptionally long-lived animals can famously regrow limbs and even complex organs and are also markedly cancer-resistant. Yun explores the cellular and molecular basis for these traits and probes the tantalizing mechanistic connections between these abilities and their extraordinary longevity and negligible senescence. Meanwhile, Steiner (Steiner 2021) examined cell senescence in bacteria, which for a long time were thought to escape senescence by dividing into two identical “daughter” cells. It is now well known that this division is asymmetric and that bacteria such as *Escherichia coli* do indeed senesce. Steiner reviews progress into bacterial senescence and highlights that aging trajectories are sensitive to environmental conditions and genotypic variation that make a mechanistic understanding elusive. Nevertheless, they optimistically point out that rapid developments in molecular toolkits and microfluidic techniques open the path to exciting opportunities to understand aging more generally.

## Eco-Evolutionary Insights Into Mechanisms

Our Research Topic also includes some papers addressing aging in the wild. Aging in wild animals was for a long time neglected because individuals were thought to be eliminated by predation or starvation before age-related physiological decline could take hold. Work by Nussey and others (Nussey et al., 2008) and Jones and others (Jones et al., 2008) convincingly showed that this was not the case, and that senescence was in fact readily detectable with sufficient data. This opened the door to studies examining the eco-evolutionary aspects of senescence in the wild. In our Research Topic, the study by Pigeon and others (Pigeon et al., 2021) on bighorn sheep is an excellent example of this work. Their study asks whether patterns of senescence in reproduction and survival may be influenced by environmental conditions during early development. This is an important question because the disposable soma theory of aging (Kirkwood 1977) would suggest that variation in resource availability could influence the balance of resource allocation to the competing processes of maintenance, growth and reproduction. Such effects have been detected in other taxa before (*see* (Cooper and Kruuk 2018; Spagopoulou et al., 2020) for examples), but this study shows that although early-life conditions influence the magnitude of survival probability and reproduction in later life, they do not necessarily influence rates of senescence. Pigeon et al. also highlight the need for clear thinking on how trajectories may be influenced by early conditions. The magnitude, age at onset, and rate of senescence combine to determine the trajectory and may be hard to distinguish. Kumar and others (Kumar et al., 2021) also tackle senescence in non-canonical model species, but from a very different perspective. They use samples from 106 bird species to examine the membrane pacemaker hypothesis (MPH), which supposes that cell membranes trade off metabolic rate with oxidative damage: cells with more unsaturated membrane fatty acids have enhanced metabolism, but are prone to oxidative damage. Thus, short-lived species are expected to have a greater degree of membrane unsaturation than long-lived species. The hypothesis offers a convenient mechanistic explanation for the cross-species apparent trade-off between longevity, fecundity and metabolic rate. Ultimately, however, Kumar et al. find little general support for the MPH and, rather, suggest that long lifespans co-evolve with long-chain fatty acids independently of the degree of unsaturation.

## Evolutionary Theories Explaining Aging Mechanisms

Several papers in the collection have important implications for our understanding of the evolution of aging and lifespan. Two, in fact, present novel evolutionary theories of aging. The adaptive hitchhike model by Omotoso and others (Omotoso et al., 2021) suggests that pro-longevity mutations may arise for reasons unrelated to longevity and then hitchhike around the tree of life. In a complementary perspective, Wensink and Cohen (Wensink and Cohen 2021) propose the Danaid theory of aging: that complex organisms are often unmaintainable, i.e. structured in ways that inadvertently prevent immortality, with clade-specific chance events having large impacts on the distribution of aging, lifespan, and the underlying mechanisms. Gems and others (Gems et al., 2021) contribute additional evidence for the evolutionary theory of semelparity, showing that *C. elegans* may be considered semelparous much like salmon, but that the pathways triggering reproductive death are also active in iteroparous organisms with more limited effects. The papers by Kumar et al. (Kumar et al., 2021) and by Simons et al. (Simons et al., 2021) provide additional evidence against straightforward, linear mediation of trade-offs by two different hypothesized mechanisms. Taken together, and with the species-specific studies mentioned above, these studies start to paint a coherent picture of the evolution of aging as a much more rich and textured landscape than previously thought, one in which no mechanistic or evolutionary theory will simply explain everything, yet in which mechanisms and evolutionary forces are tightly intertwined.

## Future Perspectives

The manuscripts presented in this Research Topic bring us a small step closer to the identification and understanding of mechanisms of aging across the tree of life, but also show that there is still a lot of work to be done before we will have a full understanding of the aging process. We hope that our Research Topic will stimulate new research focusing on more non-canonical model organisms and understudied mechanisms and will lead to the development of new and improved theories of aging that, for example, also deal with currently unexplained phenomena, such as asexual reproduction and vegetative growth. We believe that the recent emergence of state-of-the-art methods, including gene editing and tracking of individual animals in the wild, and the use of more comparative approaches, taking into account eco-evolutionary and environmental contexts, will also tremendously benefit such studies. The subsequent translation of findings from (non-)canonical model organisms to humans will hopefully assist in managing the upcoming Silver Tsunami.
